# Draft Genome Sequence of Singapore *Klebsiella pneumoniae* subsp. *pneumoniae* Isolate DS32358_14, Which Contains the Carbapenemase Gene *bla*_VIM-1_

**DOI:** 10.1128/genomeA.01377-17

**Published:** 2018-01-04

**Authors:** Tse H. Koh, Nurdyana Binte Abdul Rahman, Jeanette W. P. Teo, My-Van La, Balamurugan Periaswamy, Swaine L. Chen

**Affiliations:** a Department of Microbiology, Singapore General Hospital, Singapore; b Department of Laboratory Medicine, National University Hospital, Singapore; c National Public Health Laboratory, Singapore; d Department of Medicine, National University of Singapore, Singapore; e Infectious Diseases Group, Genome Institute of Singapore, Singapore

## Abstract

We sequenced the first *bla*_VIM-1_-positive *Klebsiella pneumoniae* strain isolated in Singapore. The isolate belongs to multilocus sequence type 2542 (ST2542), and *bla*_VIM-1_ was the first gene in an integron that also contained *aacA4*, *aphA15*, *aadA1*, *catB2*, *qacEdelta1*, and *sul1*.

## GENOME ANNOUNCEMENT

Carbapenemase-producing *Enterobacteriaceae* (CPE) strains are an increasing problem in Singapore. The most common carbapenemase genes found locally are the *bla*_KPC_, *bla*_NDM_, *bla*_OXA-48_, and *bla*_IMP_ types (1). Another major carbapenemase gene in *Enterobacteriaceae* is *bla*_VIM_, which was first described in *Klebsiella pneumoniae* in Greece and remains largely confined to southern Europe (2). We describe the complete genome sequence of the first *bla*_VIM-1_-positive *Klebsiella pneumoniae* strain isolated in Singapore.

*Klebsiella pneumoniae* strain DS32358_14 was isolated in 2014 as part of an extensive screening program for CPE colonization of patients in a large teaching hospital in Singapore.

DS32358_14 genomic DNA was sheared to approximately 300 bp using a Covaris LE220 focused ultrasonicator (Covaris). A sequencing library was prepared using the NEBNext Ultra DNA library prep kit for Illumina (E7370L) and custom primers with an 8-bp barcode index, according to the manufacturer’s instructions. This library was sequenced on an Illumina HiSeq4000 machine with 2 × 150-bp reads. The data were assembled using Velvet (version 1.2.10) (3) with a minimum contig cutoff of 500 bp, scaffolded with Opera (version 1.4.1) (4), and finished with FinIS (version 0.3) (5). In total, 4,346,527 paired-end reads passed filtering, representing an approximate coverage of 200× (based on the final assembly).

The DS32358_14 assembly consists of 141 contigs, which range in size from 513 to 516,905 bp. The combined length of all contigs is 5,748,495 bp, with an *N*_50_ size of 247,263 bp and a G+C content of 57.2%. Annotation of the draft assembly was performed using the NCBI Prokaryotic Genome Annotation Pipeline (PGAAP), which predicted 5,525 protein-coding sequences, 21 rRNA genes, and 80 tRNA genes (6).

Detection of antimicrobial resistance genes and multilocus sequence typing (MLST) were performed by SRST2 analysis of the raw sequencing reads in addition to manual analysis of the assembled contigs. DS32358_14 belonged to multilocus sequence type 2542 (ST2542) (7, 8). *bla*_VIM-1_ was the first gene in an integron that also contained *aacA4*, *aphA15*, *aadA1*, *catB2*, *qacEdelta1*, and *sul1* in sequential order. The closest match on GenBank was with the sequence of an integron on plasmid pOW16C2 in a *Klebsiella pneumoniae* isolate from wastewater in Switzerland (accession number KF977034) (9). A similar integron was found in *Escherichia coli* W1058 from Spain (accession number KF856617) (10). This is classified as an In916 integron, which is typically found in Europe (2, 11). In addition, the antimicrobial resistance genes *qnrS*, *bla*_SHV-12_, and *dfrA14* were also detected in the other parts of the genome.

### Accession number(s).

The complete genome sequence of *K. pneumoniae* DS32358_14 was deposited in GenBank under the accession number PEHD00000000. The version described in this paper is version PEHD01000000. The assembly and raw Illumina reads are collected together under BioProject PRJNA414678.
